# ﻿*Stictaflakusiorum* and *S.kukwae*—two additional new species from the Neotropics (Peltigerales, Peltigeraceae)

**DOI:** 10.3897/mycokeys.114.139681

**Published:** 2025-03-04

**Authors:** Emilia Anna Ossowska, Bibiana Moncada, Robert Lücking, Emmanuel Sérusiaux, Nicolas Magain

**Affiliations:** 1 Department of Plant Taxonomy and Nature Conservation, Faculty of Biology, University of Gdańsk, Wita Stwosza 59, PL-80-308 Gdańsk, Poland University of Gdańsk Gdańsk Poland; 2 Licenciatura en Biología, Universidad Distrital Francisco José de Caldas, Cra. 4 No. 26D-54, Torre de Laboratorios, Herbario, Bogotá D.C., Colombia Universidad Distrital Francisco José de Caldas Bogotá Colombia; 3 Research Associate, Science & Education, The Field Museum, 1400 South Lake Shore, Chicago, IL 60605, USA The Field Museum Chicago United States of America; 4 Botanischer Garten und Botanisches Museum Berlin, Freie Universität Berlin, Königin-Luise-Straße 6–8, 14195 Berlin, Germany Freie Universität Berlin Berlin Germany; 5 Evolution and Conservation Biology, University of Liège, Sart Tilman B22, 4000 Liège, Belgium University of Liège Liege Belgium

**Keywords:** Bolivia, diversity, integrative taxonomy, ITS rDNA, Lobarioideae, Peltigeraceae, Peru

## Abstract

Two additional species of *Sticta* are described as new to science based on material from Bolivia and Peru and supported by phylogenetic analysis of the fungal ITS barcoding marker. The two new species represent lineages within clade I on the global *Sticta* phylogeny. *Stictaflakusiorum* Ossowska, B. Moncada & Lücking is a species in the *S.humboldtii* morphodeme and is characterized by lobes partly to entirely covered with white hairs, also covering the margins of submarginal and laminal apothecia, and the scabrid basal membrane of cyphellae, which is white to yellow, or partly brown, and when yellow K+ purple. The taxon was discovered at a single locality in Bolivia, but it is closely related to a potentially new *Sticta* species from Peru, which is here left undescribed. The other new species, *S.kukwae* Ossowska, Magain & Sérus., belongs to the *S.weigelii* morphodeme. It has lobes with sinuous margins and dark, palmate to corymbose phyllidia. It was collected at several locations in Peru and a single locality in Bolivia.

## ﻿Introduction

Lobarioid lichens, long treated in their own family, Lobariaceae (Moncada et al. 2013a), are now recognized as a subfamily, Lobarioideae, within Peltigeraceae, along with subfamilies Nephromatoideae and Peltigeroideae (Kraichak et al. 2018; Lücking 2019; Lumbsch and Leavitt 2019; Widhelm et al. 2019, 2021). Traditionally, the presence or absence of regular pores in the lower cortex, called cyphellae, was the main feature that differentiated the genus *Sticta* (Schreb.) Ach. from other genera in the family (Galloway and Elix 2013; Moncada et al. 2013a); however, apart from *Sticta*, a second lineage, nested within the *Lobaria* clade and separated in the genus *Dendriscosticta* B. Moncada & Lücking, also features cyphellae (Moncada et al. 2013a; Simon et al. 2022). In addition to *Dendriscosticta*, another nine genera have recently been segregated from the collective genera *Lobaria* (Schreb.) Hoffm., *Pseudocyphellaria* Vain., and *Sticta*, including, e.g., *Yarrumia* D.J. Galloway (Galloway 2015) and *Emmanuelia* Ant. Simon, Lücking & Goffinet (Simon et al. 2020). Currently, close to 500 species have been accepted in the subfamily Lobarioideae (Kirk et al. 2008; Lücking et al. 2017), almost half of them within the genus *Sticta* (Moncada et al. 2014a, 2021a; Lücking et al. 2017; Ossowska et al. 2022a). The species of this genus are common in humid, cool to warm environments with high rainfall or humidity and are most diverse in tropical areas (Moncada 2012; Moncada et al. 2014b, 2020). This is illustrated by the example of Colombia, where more than 150 *Sticta* species have been identified following extensive field and laboratory work (Moncada 2012; Moncada and Lücking 2012; Moncada et al. 2013b, 2014a, b, 2015, 2021b). In comparison, the knowledge on the genus *Sticta* in neighboring countries is limited. For Ecuador, 46 names have been listed (Consortium of Lichen Herbaria 2023; Yánez-Ayabaca et al. 2023), but at least eight of these are doubtful records, including some known New Zealand endemics. Twenty-two species are included in the revised checklist for Brazil (Aptroot 2002; Dal Forno et al. 2018; Torres et al. 2021); however, Torres et al. (2021) suggest that the Cerrado forest ecoregion may host a significant number of novel *Sticta* species, indicating that the true diversity of species in Brazil may be higher. The checklist of lichens for Peru includes only ten *Sticta* species (Ramos 2014), but the majority of the records are historical and have not been critically checked. Meanwhile, twenty-eight *Sticta* taxa are known from Bolivia, mostly based on recent works (Moncada and Lücking 2012; Ossowska 2021; Ossowska et al. 2022a, b, 2024a, b; Crous et al. 2023).

This paper presents two additional new *Sticta* species, *S.flakusiorum* and *S.kukwae*, both supported by molecular data, from Peru and/or Bolivia. *Stictaflakusiorum* has been found so far at a single site in Bolivia, whereas *S.kukwae* has been collected from several localities in Peru and Bolivia. Detailed morphological and anatomical descriptions of both species are also given, together with a discussion on similar taxa.

## ﻿Materials and methods

### ﻿Taxon sampling

Fresh material for this study was collected during fieldwork in Bolivia in 2010–2017 and Peru in 2012. The collected material is deposited in the LPB, UGDA, and DUKE herbaria. All material was examined under a dissecting and a compound microscope (Nikon SMZ800N and ZEISS Axioskop). Character assessment was based on the morphological and anatomical traits for *Sticta* described by Moncada (2012) and Moncada et al. (2014a). Spot reactions were done with K (potassium hydroxide solution), C (sodium hypochlorite solution), Pd (paraphenylenediamine), and KC (K followed by C) on close spots of exposed medulla of the same thallus fragments; secondary compounds were analyzed using the thin-layer chromatography method (TLC) in solvents A and C (Orange et al. 2001).

Species that were informally distinguished by Moncada (2012) and Moncada et al. (2020) but have not yet been formally described are marked with quotes (e.g., ‘*S.arachnosylvatica*’).

### ﻿DNA extraction, PCR amplification, and sequencing

Genomic DNA from the Bolivian samples was isolated, and the nuITS rDNA marker was amplified following the protocol described in Ossowska et al. (2022a). Sequencing was performed in a Macrogen sequencing system (http://www.macrogen.com). In the case of samples from Peru, DNA was extracted following the protocol of Cubero et al. (1999). PCR conditions and primers were the same as in Ossowska et al. (2022a).

### ﻿Alignment and sequence analyses

The newly generated sequences were compared with available data from the genus *Sticta* (Suppl. material 1), using our previous alignment (Ossowska et al. 2022a) based on a recent master alignment (Moncada et al. 2020). The new sequences were added to the existing alignment using MAFFT 7.164 with the “—add” option (Katoh and Frith 2012; Katoh and Standley 2013), followed by manual checking in BIOEDIT 7.0.9 (Hall et al. 2011). Phylogenetic analysis was performed using maximum likelihood in RAxML 8.2.0 (Stamatakis 2014) on the CIPRES Science Gateway (Miller et al. 2010), with non-parametric bootstrapping using 400 pseudoreplicates (based on an automated saturation criterion) under the universal GTR GAMMA model. Trees were visualized in FigTree 1.4.2 (Drummond and Rambaut 2007) and edited using Coral Draw 2019.

## ﻿Results

**Figure 1.**
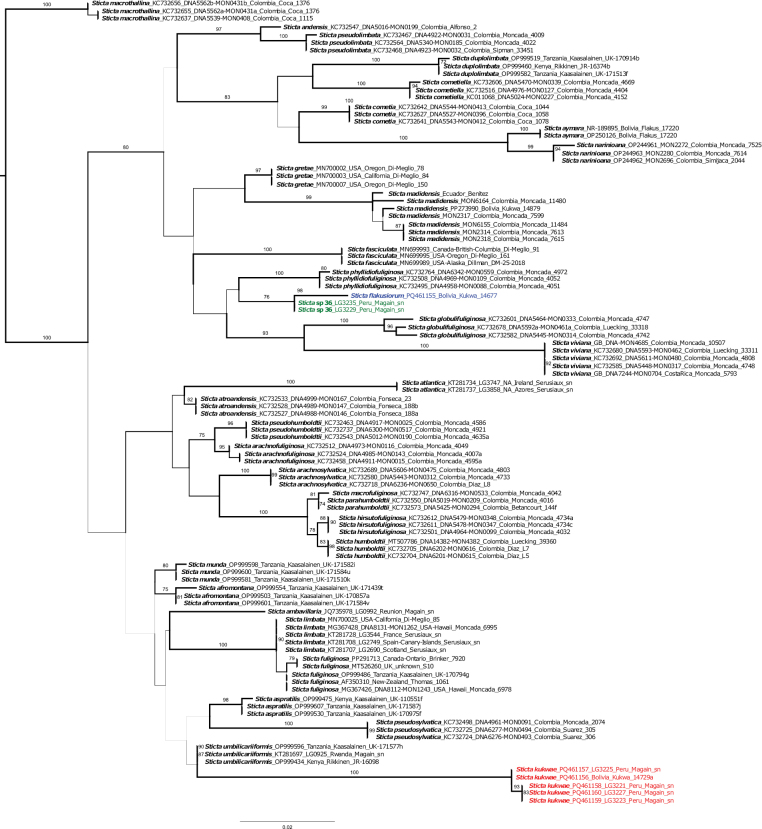
Best-scoring maximum likelihood tree of the *Sticta* target clade containing the new species *S.flakusiorum* from Bolivia (blue), *S.kukwae* from Bolivia and Peru (red), and *S.* sp. 36 from Peru (green), based on the fungal ITS barcoding marker. Branches associated with high bootstrap support values (≥ 70) are thickened and values are indicated near the branches.

We generated seven new nuITS rDNA sequences that form two distinct lineages in the *Sticta* tree (Fig. 1), suggesting the presence of three new species, two of which are closely related sister species. The new sequences align close to other *Sticta* species, such as *S.sylvatica* (Huds.) Ach. and *S.aymara* Ossowska et al., within clade I (*fuliginosa* clade) sensu Widhelm et al. (2018).

The first of the new species, *S.kukwae*, is represented by one specimen from Bolivia and four from Peru. All specimens have a thallus with strongly sinuous margins and very dark phyllidia. One specimen (LG3227) from Peru had small and sparse apothecia, absent in the other specimens. However, other characteristics were consistent with the rest of the specimens in this clade (Fig. 1). Within clade I, the new species is closely related to *S.umbilicariiformis* Hochst. ex Flot. (Fig. 1).

The second new lineage is formed by two sister species. The lineage with a specimen from Bolivia is named in this paper *S.flakusiorum* and is closely related to the specimens from Peru, which potentially also represent a new species. At this point, we have named it *Sticta* sp. 36. It is a phyllidiate species (*S.flakusiorum* lacks vegetative diaspores), and morphologically it is similar to *S.phyllidiokunthii* B. Moncada & Lücking, with numerous, aggregated, palmate phyllidia that are marginal and laminal in *S.* sp. 36 and marginal in *S.phyllidiokunthii* (Moncada et al. 2013b). However, the material of *S.* sp. 36 is too sparse for a formal description at this point. Both species, *S.flakusiorum* and *S.* sp. 36, are nested in the clade of *S.viviana* Alej. Suárez & Lücking and are closely related to *S.phyllidiofuliginosa* B. Moncada, A. Suárez & Lücking.

Detailed descriptions of morphological and anatomical characteristics of *S.flakusiorum* and *S.kukwae*, together with figures and comparisons with similar and related species, are given below.

## ﻿Discussion

As a genus, *Sticta* is relatively easy to recognize in the field due to the foliose, large thallus with tomentum and cyphellae on the lower surface and the often characteristic fishy odor caused by the presence of methylamine products (Galloway 1994, 1997; Moncada 2012). However, species within this genus are much more difficult to distinguish, due to the lack of a clear concept of within-species variation, including the type of photobiont, the presence and type of vegetative propagules, their shape, distribution, and size, as well as the width and length of the lobes or their shape (Moncada 2012). Molecular data have helped to address this issue and to refine the set of potentially diagnostic characters, and Moncada et al. (2014a) identified a total of over 150 morphological and anatomical features that should be taken into account (see also Ossowska et al. 2024a).

Even so, accurate species recognition may be hindered by intraspecific variability, such as the formation of photosymbiodemes, apotheciate vs. non-apotheciate species pairs, and discrete morphodemes (Moncada et al. 2020, 2021b; Ossowska et al. 2022a, b, 2024a; Di Meglio and Goward 2023). For instance, the two vegetatively reproducing *S.fuliginosa* (With.) Ach. (with isidia) and *S.limbata* (Sm.) Ach. (with soredia) cannot be distinguished using the ITS barcoding marker (Moncada et al. 2014a; Magain and Sérusiaux 2015). Another case is found in the large foliose *S.filix* (Sw.) Nyl. vs. the delicate *S.lacera* (Hook. f. & Taylor) Müll. Arg., both New Zealand endemics (Lücking et al. 2022), or in *S.antoniana* B. Moncada & Lücking and *S.tomentosa* (Sw.) Ach. from Hawaii (Moncada et al. 2020, 2021a), as well as *S.arenosella* Di Meglio & Goward and *S.torii* Ant. Simon & Goward (Simon et al. 2018a; Di Meglio and Goward 2023; Ossowska et al. 2024a, b).The *Stictafuliginosa* clade also contains apotheciate specimens devoid of isidia or soredia, suggesting the existence of individuals within the same species with different modes of reproduction. This was also observed in our new species, *S.kukwae*, with apothecia in one specimen from Peru, arranged in a clade together with non-apotheciate specimens. Similar cases were recently reported for *S.scabrosa* B. Moncada, Merc.-Díaz & Bungartz subsp.scabrosa and *S.cellulosa* Kaasalainen, originally described as sterile (Moncada et al. 2021b; Kaasalainen et al. 2023), but later found fertile in material from Bolivia (Ossowska et al. 2022b, 2024b). Some species, e.g., the widely distributed *S.andina*, show even greater variation in reproduction modes: this species was previously divided into three tentative taxa that differed in the type of propagation (Moncada et al. 2021a, b; Ossowska et al. 2022a; Kaasalainen et al. 2023), but later they were merged into one species due to genetic similarities (Moncada et al. 2021b).

Traditional taxonomy in *Sticta* was largely based on morphodemes, i.e., particular gross morphologies that were recognized as species, e.g., narrow-lobed, cyanobacterial individuals with marginal isidia as *S.weigelii* (Ach.) Vain., broad-lobed, cyanobacterial specimens with laminal isidia as *S.fuliginosa*, or green-algal, apotheciate individuals as *S.canariensis* (Bory) Bory ex Delise, *S.damicornis* (Sw.) Ach., or *S.dichotoma* Bory ex Delise. Molecular data have shown that these morphodemes consist of many, often only distantly related species (Moncada et al. 2013b, 2014a, 2015; Lücking et al. 2021; Ossowska et al. 2024a). The two additional new species of *Sticta* introduced in this paper are also part of morphodemes: *S.kukwae* is morphologically similar to *S.weigelii*, both having a brown, irregular to orbicular thallus with dark, marginal vegetative propagules, whereas *S.flakusiorum*, due to its hairy upper surface, represents the *S.humboldtii* morphodeme.

In recent years, research on *Sticta* has intensified, resulting in the addition of many new species in various parts of the world (e.g., Lendemer and Goffinet 2015; Magain and Sérusiaux 2015; Simon et al. 2018a, b; Dal Forno et al. 2018; Torres et al. 2021; Ossowska et al. 2022a, 2024a; Kaasalainen et al. 2023; Di Meglio and Goward 2023; Yánez-Ayabaca et al. 2023). Many regions in the Neotropics, e.g., Colombia, Bolivia (Ossowska et al. 2024a) and Puerto Rico (Mercado-Díaz et al. 2020), but also Africa (Simon et al. 2018b; Kaasalainen et al. 2023) and Oceania, e.g., Hawaii (Moncada et al. 2020, 2021a), are being explored, but many other regions remain poorly studied in terms of the genus *Sticta*. For instance, the checklist of lichens from Peru includes only ten *Sticta* species (Ramos 2014), and these have not yet been critically restudied. Among the names in the Peruvian checklist are *S.fuliginosa*, *S.laciniata* Ach., *S.sylvatica*, and *S.weigelii*, in which many new species have recently been distinguished (Moncada et al. 2021b; Di Meglio and Goward 2023; Ossowska et al. 2024a; this study). The new species presented here, *S.kukwae*, and the as-yet-undescribed *S.* sp. 36, are the first specimens from Peru supported by molecular data.

### ﻿Taxonomy

#### ﻿ Sticta flakusiorum

Taxon classificationFungiPeltigeralesPeltigeraceae

Ossowska, B. Moncada & Lücking sp. nov.

D873FD1C-3E20-5FCD-89BC-FD3D0D4B9EFA

856228

Fig. 2

##### Diagnosis.

Differing from *S.humboldtii* in the absence of true cilia, the presence of submarginal apothecia with entire to crenate margins, completely to partly covered by white hairs, spongy to fasciculate primary tomentum, and scabrid basal membrane of cyphellae, white to yellow (then K+ purple), or partly brown.

**Figure 2.**
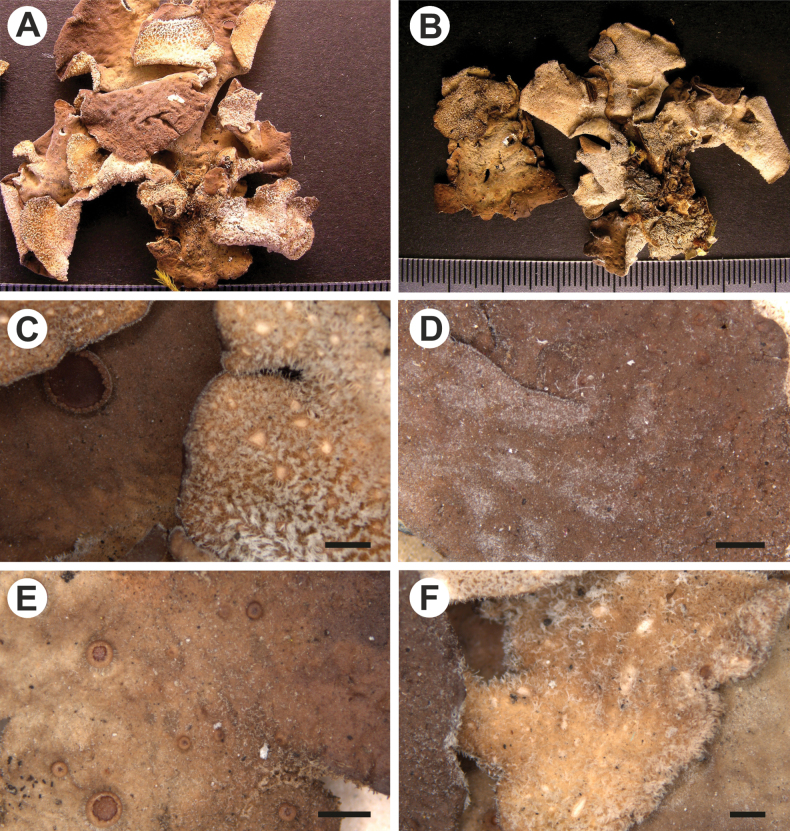
Morphology of *Stictaflakusiorum* (holotype) **A** upper surface **B** lower surface **C, D** hirsute upper surface with apothecium and lower surface with tomentum and cyphellae **E** apothecia with entire to crenate margins, covered by white hairs **F** primary tomentum spongy to fasciculate and cyphellae with scabrid basal membrane. Scale bars: 1 mm (**A–F**).

##### Type.

Bolivia. • Dept. La Paz; Prov. Bautista Saavedra, Área Natural de Manejo Integrado Nacional APOLOBAMBA, between La Curva and Charazani, 15°08'09"S, 69°02'03"W, 3780 m, open area with shrubs, Ceja de Monte Superior (Altimontano), on shrub, 15 Nov. 2014, M. Kukwa 14677 (holotype UGDA L-65223, isotype LPB).

##### Description.

Stipe absent. Thallus orbicular, up to 5 cm diam., moderately branched, with 3–5 branches per 5 cm radius, branching pleurotomous to polytomous; lobes suborbicular to flabellate, interspaced to adjacent, involute, with their apices rounded, revolute, and undulate and their margins sinuous, slightly thickened; lobe internodes 2–20 mm long, 3–15 mm broad; thallus coriaceous. Upper surface pitted to rugose, yellowish brown to chocolate brown, darker near the apices in the herbarium, shiny; lobes entirely hirsute or rarely with some parts lacking tomentum, covered by white hairs, without papillae and maculae; true cilia absent, but lower tomentum partly projecting beyond the margins and resembling cilia, fasciculated to agglutinated, white to pale brown, up to 0.5 mm. Apothecia submarginal and laminal, subaggregated, sessile to shortly stipitate, with pronounced invagination on the lower side, up to 2.0 mm diam.; disc brown to chestnut-brown; margin entire to crenate, completely to partly covered by white hairs, up to 1 mm long, simple to agglutinated, margin brown to dark brown. Vegetative propagules absent. Lower surface ribbed, brown; primary tomentum dense and usually thick to sparse to the margin, spongy to fasciculate, soft, white to brown; secondary tomentum present, arachnoid. Rhizines absent. Cyphellae 1–20 per cm^2^ towards the thallus center and 41–60 per cm^2^ towards the margin, scattered, elongate to irregular, urceolate with wide pore to cupuliform, erumpent to sessile, remaining below the level of the primary tomentum, with the margin raised and involute to erect, cream to brown colored, with tomentum up to the pore; pore up to 1.5 mm diam.; basal membrane scabrid, white to yellow, or partly brown, when yellow K+ purple and C+ red-orange, KC–, P–. Medulla compact, white to yellow, or partly brown, when yellow K+ purple and C+ red-orange, KC–, P–. No substances detected by TLC.

Upper cortex paraplectenchymatous, up to 35 μm thick, uniform, up of 5 layers of cells, their walls up to 1.5 μm thick and their lumina rounded to isodiametric, up to 5–15 × 5–10 μm diam. Photobiont layer up to 150 μm thick, its cells up to 10 μm diam. Medulla up to 120 μm thick, its hyphae up to 5.0 μm broad. Lower cortex paraplectenchymatous, up to 50 μm thick, with up to 7 cell layers; cells up to 10 μm diam. Upper primary tomentum up to 100 μm long, simple or in fascicles formed of up to 7 hyphae, hyphae simple. Upper secondary tomentum not seen on upper surface. Lower primary tomentum up to 200 μm long, composed of fascicles formed of 10–15 hyphae, hyphae mostly simple, apically free, and flexuous. Lower secondary tomentum 30 μm long, of single, simple to branched hairs, moniliform. Cyphellae cavity up to 220 μm deep; cells of basal membrane without or rarely with up to 2 papillae. Apothecia biatorine, up to 500 μm high, with indistinct stipe, about 20 μm high; excipulum up to 400 μm broad, with projecting hairs, up to 1 µm long. Hymenium up to 300 μm high; epihymenium up to 5 μm high, orange-brown, pigment present in the gel and in the walls upper cells of paraphyses, with very gelatinous upper layer. Asci 4–8-spored, ascospores fusiform, 1–3-septate, 25–35 × 6–8 μm.

##### Habitat and distribution.

*Stictaflakusiorum* is an epiphytic species found in an open area with shrubs at an altitude of 3780 m in the Department La Paz, Bolivia.

##### Etymology.

The species is named in honor of two lichenologists, Adam Flakus and Pamela Rodriguez-Flakus, for their contributions to the taxonomy of lichens and lichenicolous fungi of Bolivia.

##### Notes.

The new species, *S.flakusiorum*, forms part of the *S.humboldtii* morphodeme, which also includes *S.pseudohumboldtii* B. Moncada & Lücking and *S.parahumboldtii* B. Moncada & Lücking (Moncada et al. 2013b). However, unlike in the other species, the upper surface of *S.flakusiorum* is rather hirsute, while in *S.humboldtii* and the other species, the hairs are very dense and resemble the primary tomentum present on the lower surface (Moncada 2012). In addition, *S.parahumboldtii* has marginal soredia and lacks apothecia (Moncada et al. 2013b). Furthermore, all species differ in the color of the lower surface and tomentum. In the new species, the lower surface is brown, and the primary tomentum is white to cream. Other species have a cream-colored lower surface, and the primary tomentum is cream in *S.parahumboldtii*, cream-white in *S.pseudohumboldtii*, and cream to grey-brown in *S.humboldtii* (Moncada 2012; Moncada et al. 2013b). All species belong to clade I on the *Sticta* phylogeny (see Fig. 1), but the new species is more closely related to *S.viviana*. *Stictahumboldtii* and *S.parahumboldtii* are related to ‘*S.arachnosylvatica*’, while *S.pseudohumboldtii* is close to *S.arachnofuliginosa* B. Moncada & Lücking (Widhelm et al. 2018). Among the species of this morphodeme, *S.humboldtii* has been reported more frequently than other species (Moncada 2012; Moncada et al. 2013b), including records from Peru (Ramos 2014). However, only the Colombian records are supported by molecular data (Moncada et al. 2013b, 2014a), and therefore its presence in Peru needs to be verified. *Stictapseudohumboldtii* and *S.parahumboldtii* are known so far only from Colombia (Moncada 2012; Moncada et al. 2013b, 2014a, b).

In the phylogenetic tree, *S.flakusiorum* forms a lineage sister to a clade of a potentially new species, referred to as *Sticta* sp. 36 (see above). This taxon is distinguished by its thallus with smooth upper surface, sparse and laminal apothecia, and abundant, marginal phyllidia. Furthermore, the primary tomentum is greyish gold, whereas in *S.flakusiorum* it is white to brown. The specimens of *S.* sp. 36 are fragmentary; thus, we have decided not to describe it at this moment. *Sticta* sp. 36 was found in Peru in Puno (Lampa, Santa Lucia).

The hirsute upper surface is also characteristic of ‘*S.arachnosylvatica*’, *S.minutula* B. Moncada, A. Suárez & Lücking and *S.hirta* (Nyl.) Trevis (Moncada 2012; Moncada et al. 2014a, 2020), but these taxa differ from *S.flakusiorum* in the structure of the lobes, the presence of vegetative propagules, as well as the color of the lower surface and the structure of primary tomentum. In particular, the lobe margins in all these species are entire to crenate, whereas in *S.flakusiorum* they are sinusoidal; in addition, ‘*S.arachnosylvatica*’ and *S.minutula* have isidia. The lower surface of ‘*S.arachnosylvatica*’ is cream-white with primary tomentum dense to the margin (Moncada 2012), and in *S.minutula* the lower surface is cream-white with primary tomentum scarce over the whole area. Additionally, the latter taxon is distinguished by its sparse cyphellae (Moncada 2012). *Stictahirta* has a creamy lower surface with irregular tomentum, sparse towards the margins, and it is fasciculate to spongy (Moncada 2012). All three species, ‘*S.arachnosylvatica*,’ *S.minutula*, and *S.hirta*, have been molecularly confirmed only from Colombia (Moncada 2012; Moncada et al. 2014a, 2020) but have not been reported from Bolivia and Peru.

#### ﻿ Sticta kukwae

Taxon classificationFungiPeltigeralesPeltigeraceae

Ossowska, Magain & Sérusiaux sp. nov.

1359B908-F392-529F-9EAE-6F44A12EC33B

856229

Fig. 3

##### Diagnosis.

Differing from *S.weigelii* in lobes with sinuous margins, in the presence of marginal phyllidia, and the scarce, submarginal apothecia, as well as the primary tomentum being light brown to brown, dense, and sparse towards the margins.

**Figure 3.**
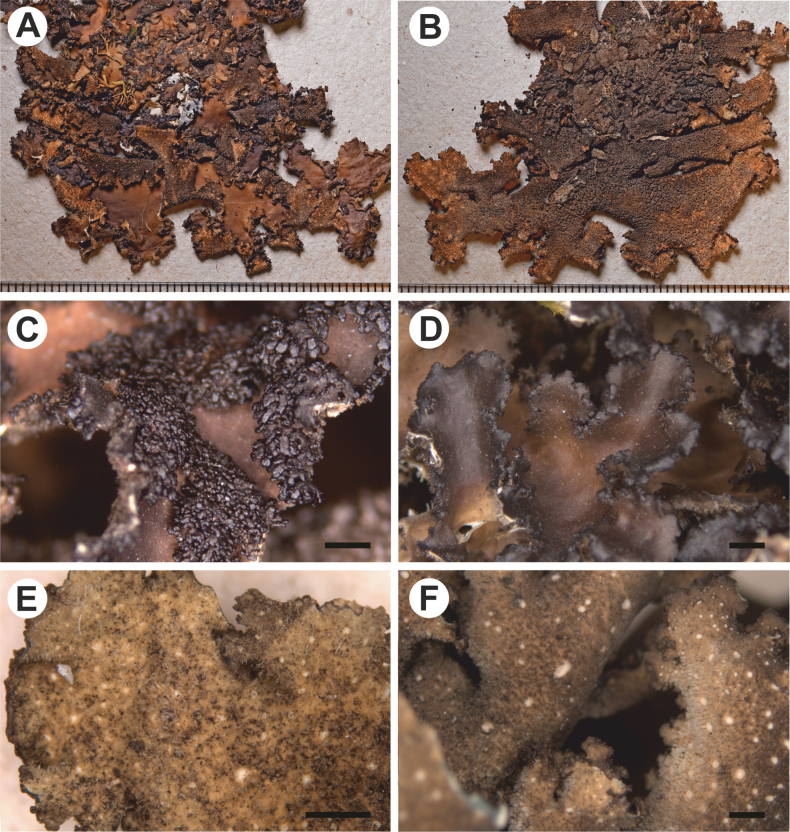
Morphology of *Stictakukwae* (**A, B, E** holotype **C** LG3223, **D** LG3221, **F** LG3227) **A** upper surface **B** lower surface **C, D** lobes with sinuous margins and marginal phyllidia **E** lower tomentum with cyphellae and rhizines. Scale bars: 1 mm (**A–F**).

##### Type.

Bolivia. • Dept. La Paz; Prov. Franz Tamayo, Área Natural de Manejo Integrado Nacional APOLOBAMBA, between la Cumbre and Pelechuco, close to Aguas Blancas, 14°49'12"S, 69°07'05"W, elev. 4070 m, open high Andean vegetation, Altoandino, saxicolous, 15 Nov. 2014, M. Kukwa 14729a (holotype UGDA L-65224, isotype LPB).

##### Description.

Stipe absent. Thallus suborbicular to irregular, up to 10 cm diam., moderately branched, with 3–5 branches per 5 cm radius, branching anisotomous to polytomous; lobes ligulate to flabellate, undulate, with their apices rounded, revolute, and their margins sinuous, not thickened; lobe internodes 5–9 mm long, 4–9 mm broad; thallus coriaceous. Upper surface smooth to shallowly pitted, yellowish brown to brown when dry, shiny; surface glabrous, few lobes with papillae but without maculae; true cilia absent. Only two apothecia found, submarginal, with slightly pronounced invagination on lower side, up to 1.5 mm diam.; disc brown; margin smooth, brown to dark brown. Phyllidia present, marginal and laminal, simple, branched, palmate to corymbose, vertical to obliquely arranged, globular at first, then spatulate to squamiform, usually darker than the thallus. Lower surface uneven, light brown; primary tomentum dense and thick to the margin, sometimes absent at the very edge, fasciculate to spongy, soft, white to brown, sometimes brown with brighter apices; secondary tomentum present, arachnoid. Rhizines present, only on few lobes, whitish to brown, simple to branched, densely distributed. Cyphellae 1–20 per cm^2^ towards the thallus center and 1–20 per cm^2^ towards the margin, dispersed, rounded to irregular, urceolate with wide pore, erumpent to sessile, remaining below the level of the primary tomentum, with the margin elevated and involute, white to beige colored, with tomentum; pore up to 0.5 mm diam.; basal membrane scabrid, white, K+ yellowish, C–, KC–, P–. Medulla compact, white, K–, C–, KC–, P–. No substances detected by TLC.

Upper cortex paraplectenchymatous, up to 65 μm thick, uniform, consisting of up to 7 cell layers with cells 5–10 μm diam., their walls up to 1.5 μm thick. Photobiont layer up to 130 μm thick, its cells up to 15 μm diam. Medulla up to 120 μm thick, its hyphae 4 μm broad, without crystals. Lower cortex paraplectenchymatous, up to 60 thick, with 7 cell layers; cells up to 10 μm diam., their walls up to 2.5 μm thick. Lower primary tomentum up to 400 μm long, with cells resembling secondary tomentum and probably representing thalloconidia, simple or in fascicles formed of up to 20 hyphae, hyphae simple. Lower secondary tomentum 70 mm long, simple to branched, moniliform. Cyphellae cavity up to 150 μm deep; cells of basal membrane without or with single papillae. Apothecia lecanorine (with algal layer below cortex), up to 250 μm high, without distinct stipe; excipulum 150 μm broad, without projecting hairs. Hymenium up to 75 μm high; epihymenium 5 μm high, orange-brown with gelatinous upper layer. Asci immature. Ascospores not observed.

##### Habitat and distribution.

*Stictakukwae* is known from Bolivia and Peru. In Bolivia, it was found saxicolous and was collected at a single locality in the Área Natural de Manejo Integrado Nacional Apolobamba in the Department La Paz, at an altitude of 4070 m. In Peru, it was also saxicolous and found in four localities in Puno, in a vegetation type of Roquedal, Matorral de Puna, at an altitude of 3850 m.

##### Etymology.

Named in honor of the lichenologist Martin Kukwa for his contribution to the taxonomy of lichens and lichenicolous fungi in Bolivia.

##### Additional material examined.

Peru. • Puno - Carabaya, Ollachea - Macusani (20 km of Macusani), in a vegetation type of Roquedal, Matorral de Puna, on rocks on the ground/close to the ground, 23 May 2012, N. Magain (LG3225, LG3227, LG3221 & LG3223).

##### Notes.

*Stictakukwae* is another species in the *S.weigelii* morphodeme, along with the recently described *S.andina* B. Moncada, Lücking & Sérus., *S.scabrosa*, and *S.waikamoi* Moncada & Lücking. It differs from these species in the type of vegetative propagules and the presence of lobes with strongly sinuous margins, which have not been observed in the other species. *Stictaweigelii* s.str. and *S.waikamoi* produce isidia, and *S.andina* has isidia and phyllidia. *Stictascabrosa*, as *S.kukwae*, produces phyllidia, but in this taxon, they are the same color as the thallus, whereas in the new species, they are blackish-brown. Both species can produce sparse apothecia, but in *S.kukwae* their margins are crenate and dark brown, whereas in *S.scabrosa* they are entire to very rarely shallowly crenate and in the same color as the thallus (Moncada et al. 2021b; Ossowska et al. 2022b). *Stictaandina* may also have apothecia, but they are abundant and with verrucose to crenate margins (Moncada et al. 2021a, b; Ossowska et al. 2022b). Another difference is found in the color of the lower surface, as *S.andina* has dark lower surface, in *S.scabrosa* it is yellow-brown, while in *S.weigelii* the color ranges from beige to dark brown, and in *S.waikamoi* it is dark brown (Moncada et al. 2020, 2021a, b; Ossowska et al. 2022b). The newly described species has a light brown lower surface. *Stictaandina* and *S.scabrosa* have a wide distribution (Moncada et al. 2021a, b; Kaasalainen et al. 2023). In contrast, *S.weigelii* was previously assumed to be widespread (Galloway 1994, 1997, 2006). However, recent research has shown that its distribution is probably limited to the Neotropics (Moncada et al. 2021b; Mercado-Díaz et al. 2023). All three taxa are also known from Bolivia (Ossowska 2021; Ossowska et al. 2022b). *Stictawaikamoi* is known from the Hawaiian islands (Moncada et al. 2020, 2021a). Only *S.weigelii* has been reported from Peru (Ramos 2014), but without molecular evidence.

In the phylogenetic tree (Fig. 1), the new species is closely related to *S.umbilicariiformis*. However, it has many marginal pustules, which can sometimes make it appear sorediate; thalli is often quite large, and lobes are thick with wavy to foveolate margins. Additionally, the lower surface is cream-colored to brown and thickly tomentose. *Stictaumbilicariiformis* has been documented in East Africa, with a high probability of its occurrence in other regions as well (Magain and Sérusiaux 2015; Kaasalainen et al. 2023).

The presence of lobes with sinuous margins is also a characteristic feature in the recently distinguished *S.monlueckiorum* Ossowska, Flakus & Rodr.-Flakus from Bolivia. In *S.monlueckiorum*, the thallus is larger (up to 10 cm) and moderately branched, while the apothecia are laminal with hirsute margins and without vegetative propagules (Crous et al. 2023), whereas *S.flakusiorum* has a hirsute upper surface with abundant, submarginal apothecia and without vegetative propagules. All three taxa differ also in the color of the lower surface and the density of the cyphellae. In *S.monlueckiorum*, the lower surface is beige to yellowish, and the cyphellae have a density of 41–60 per cm^2^ towards the center and more than 100 towards the margins (Crous et al. 2023). In *S.flakusiorum*, the lower surface is brown, and the cyphellae are 1–20 per cm^2^ towards the center and 41–60 per cm^2^ towards the margins, and in *S.kukwae*, 1–20 per cm^2^ towards the thallus center and margins.

The hyphae of primary tomentum of *Stictakukwae* produce peculiar structures that resemble budding conidia forming chains. Similar structures were found in the isidiate *S.atlantica* Magain & Sérus., *S.fuliginoides* Magain & Sérus., and *S.fuliginosa* by Magain and Sérusiaux (2015), who stated in the case of *S.fuliginosa* they can act as conidia. These cells in the mentioned species are very similar to cells of secondary tomentum in several *Sticta* species, and possibly both can play a role of conidia. Such spores thus can be named thalloconidia, which are on the other hand known mainly in several species of the genus *Umbilicaria* Hoffm. (Hestmark 1990, 1991, 1992), but also in some crustose lichens (e.g., *Miriquidicanephaea* (Sommerf.) P.F. Cannon, *Protoparmelialeproloma* (R. Sant.) Rambold & Poelt, *Protoparmeliopsispeltata* (DC.) Arup, Zhao Xin & Lumbsch, *Rhizoplacamelanophthalma* (DC.) Leuckert & Poelt, *Sporastatiakarakorina* (Obermayer & Poelt) Davydov & Yakovch.) (Poelt and Obermayer 1990). However, the ultrastructural study of their development must be performed prior to the final change in the conception of their role.

## Supplementary Material

XML Treatment for Sticta flakusiorum

XML Treatment for Sticta kukwae

## ﻿Additional information

### Author ORCIDs

Emilia Anna Ossowska https://orcid.org/0000-0002-1357-6071

Bibiana Moncada https://orcid.org/0000-0001-9984-2918

Robert Lücking https://orcid.org/0000-0002-3431-4636

Emmanuel Sérusiaux https://orcid.org/0000-0002-0456-0131

Nicolas Magain https://orcid.org/0000-0001-5409-9518

### Data availability

All of the data that support the findings of this study are available in the main text or Supplementary Information.

## Supplementary materials

Supplementary material 1 Specimens of *Sticta* used in molecular analysis with locality, voucher information, GenBank accession numbers and list of references Data type xlsx Explanation note Sequences generated for this study are in bold. https://binary.pensoft.net/file/1269320This dataset is made available under the Open Database License (http://opendatacommons.org/licenses/odbl/1.0/). The Open Database License (ODbL) is a license agreement intended to allow users to freely share, modify, and use this Dataset while maintaining this same freedom for others, provided that the original source and author(s) are credited.Emilia Anna Ossowska, Bibiana Moncada, Robert Lücking, Emmanuel Sérusiaux, Nicolas Magain

## References

[B1] Aptroot A (2002) New and interesting lichens and lichenicolous fungi in Brazil. Fungal Diversity 9: 15–45.

[B2] Consortium of Lichen Herbaria (2023) Consortium of Lichen Herbaria – building a Global Consortium of Bryophytes and Lichens as keystones of cryptobiotic communities. [WWW resource] https://lichenportal.org/ [Accessed 5 March 2023]

[B3] Crous PW Costa MM Kandemir H Vermaas M Vu D Zhao L Arumugam E Flakus A Jurjević Ž Kaliyaperumal M Mahadevakumar S Murugadoss R Shivas RG Tan YP Wingfield MJ Abell SE Marney TS Danteswari C Darmostuk V Denchev CM Denchev TT Etayo J Gené J Gunaseelan S Hubka V Illescas T Jansen GM Kezo K Kumar S Larsson E Mufeeda KT Piątek M Rodriguez-Flakus P Sarma PVSRN Stryjak-Bogacka M Torres-Garcia D Vauras J Acal DA Akulov A Alhudaib K Asif M Balashov S Baral H-O Baturo-Cieśniewska A Begerow D Beja-Pereira A Bianchinotti MV Bilański P Chandranayaka S Chellappan N Cowan DA Custódio FA Czachura P Delgado G De Silva NI Dijksterhuis J Dueñas M Eisvand P Fachada V Fournier J Fritsche Y Fuljer F Ganga KGG Guerra MP Hansen K Hywel-Jones N Ismail AM Jacobs CR Jankowiak R Karich A Kemler M Kisło K Klofac W Krisai-Greilhuber I Latha KPD Lebeuf R Lopes ME Lumyong S Maciá-Vicente JG Maggs-Kölling G Magistà D Manimohan P Martín MP Mazur E Mehrabi-Koushki M Miller AN Mombert A Ossowska EA Patejuk K Pereira OL Piskorski S Plaza M Podile AR Polhorský A Pusz W Raza M Ruszkiewicz-Michalska M Saba M Sánchez RM Singh R Śliwa L Smith ME Stefenon VM Strašiftáková D Suwannarach N Szczepańska K Telleria MT Tennakoon DS Thines M Thorn RG Urbaniak J van der Vegte M Vasan V Vila-Viçosa C Voglmayr H Wrzosek M Zappelini J Groenewald JZ (2023) Fungal Planet description sheets: 1550–1613. Persoonia - Molecular Phylogeny and Evolution of Fungi. 10.3767/persoonia.2023.51.08 PMC11041897 38665977

[B4] Cubero OF Crespo A Fatehi J Bridge PD (1999) DNA extraction and PCR amplification method suitable for fresh, herbarium-stored, lichenized, and other fungi. Plant Systematics and Evolution 216(3–4): 243–249. 10.1007/BF01084401

[B5] Dal Forno M Moncada B Lücking R (2018) *Stictaaongstroemii*, a newly recognized species in the *S.damicornis* morphodeme (Lobariaceae) potentially endemic to the Atlantic Forest in Brazil. Lichenologist (London, England) 50(6): 691–696. 10.1017/S0024282918000403

[B6] Di Meglio JR Goward T (2023) Resolving the *Stictafuliginosa* morphodeme (lichenized Ascomycota: Peltigeraceae) in northwestern North America. The Bryologist 126(1): 090–110. 10.1639/0007-2745-126.1.090

[B7] Drummond AJ Rambaut A (2007) BEAST: Bayesian evolutionary analysis by sampling trees. BMC Evolutionary Biology 7(1): 214. 10.1186/1471-2148-7-214 17996036 PMC2247476

[B8] Galloway DJ (1997) Studies on the lichen genus *Sticta* (Schreber) Ach. IV. New Zealand species. Lichenologist (London, England) 29(2): 105–168. 10.1006/lich.1996.0066

[B9] Galloway DJ (2006) Notes on the holotype of *Stictadamaecornis* β *weigelii* Ach. (=*Stictaweigelii*). Lichenologist (London, England) 38(1): 89–92. 10.1017/S0024282905015598

[B10] Galloway DJ (2015) Contributions to a history of New Zealand lichenology 5*. James Murray (1923–1961). Phytotaxa 198(1): 1. 10.11646/phytotaxa.198.1.1

[B11] Galloway DJ Elix JA (2013) Reinstatement of *Crocodia* Link (Lobariaceae: Ascomycota) for five species formerly included in *Pseudocyphellaria* Vain. Australasian Lichenology 72: 32–42.

[B12] Galloway DJ Southern South American Species (1994) Studies on the lichen genus *Sticta* (Schreber) Ach.: I. Southern South American species. Lichenologist (London, England) 26(3): 223–282. 10.1006/lich.1994.1019

[B13] Hall T Biosciences I Carlsbad C (2011) BioEdit: An important software for molecular biology. GERF Bulletin of Biosciences: 2.

[B14] Hestmark G (1990) Thalloconidia in the genus *Umbilicaria*. Nordic Journal of Botany 9(5): 547–574. 10.1111/j.1756-1051.1990.tb00546.x

[B15] Hestmark G (1991) Teleomorph-anamorph relationships in *Umbilicaria* II. Patterns in propagative morph production. Lichenologist (London, England) 23(4): 361–380. 10.1017/S0024282991000518

[B16] Hestmark G (1992) Conidiogenesis in five species of *Umbilicaria*. Mycological Research 96(12): 1033–1043. 10.1016/S0953-7562(09)80113-5

[B17] Kaasalainen U Kirika PM Mollel NP Hemp A Rikkinen J (2023) The Lichen Genus *Sticta* (Lobariaceae, Peltigerales) in East African Montane Ecosystems. Journal of Fungi (Basel, Switzerland) 9(2): 246. 10.3390/jof9020246 36836360 PMC9961217

[B18] Katoh K Frith MC (2012) Adding unaligned sequences into an existing alignment using MAFFT and LAST. Bioinformatics (Oxford, England) 28(23): 3144–3146. 10.1093/bioinformatics/bts578 23023983 PMC3516148

[B19] Katoh K Standley DM (2013) MAFFT multiple sequence alignment software version 7: Improvements in performance and usability. Molecular Biology and Evolution 30(4): 772–780. 10.1093/molbev/mst010 23329690 PMC3603318

[B20] Kirk PM Cannon PF Minter DW Stalpers JA (2008) Dictionary of the Fungi. 10^th^ edn. CAB International, Wallingford.

[B21] Kraichak E Huang JP Nelsen M Leavitt SD Thorsten Lumbsch H (2018) A revised classification of orders and families in the two major subclasses of Lecanoromycetes (Ascomycota) based on a temporal approach. Botanical Journal of the Linnean Society 188(3): 233–249. 10.1093/botlinnean/boy060

[B22] Lendemer JC Goffinet B (2015) *Stictadeyana*: A New Endemic Photomorphic Lichen from the Imperiled Mid-Atlantic Coastal Plain of Eastern North America. Systematic Botany 40(4): 933–941. 10.1600/036364415X689979

[B23] Lücking R (2019) Stop the Abuse of Time! Strict Temporal Banding is not the Future of Rank-Based Classifications in Fungi (Including Lichens) and Other Organisms. Critical Reviews in Plant Sciences 38(3): 199–253. 10.1080/07352689.2019.1650517

[B24] Lücking R Moncada B Soto-Medina E Simijaca D Sipman HJM (2017) Actualización nomenclatural y taxonómica del Catálogo de Liqúenes de Colombia. Revista de la Academia Colombiana de Ciencias Exactas, Físicas y Naturales 45(174): 147–189. 10.18257/raccefyn.1266

[B25] Lücking R Leavitt SD Hawksworth DL (2021) Species in lichen-forming fungi: Balancing between conceptual and practical considerations, and between phenotype and phylogenomics. Fungal Diversity 109(1): 99–154. 10.1007/s13225-021-00477-7

[B26] Lücking R Moncada B Widhelm TJ Lumbsch HT Blanchon DJ de Lange PJ (2022) The *Stictafilix* - *Stictalacera* conundrum (lichenized Ascomycota: Peltigeraceae subfamily Lobarioideae): unresolved lineage sorting or developmental switch? Botanical Journal of the Linnean Society 199(3): 706–727. 10.1093/botlinnean/boab083

[B27] Lumbsch HT Leavitt SD (2019) Introduction of subfamily names for four clades in Cladoniaceae and Peltigeraceae (Lecanoromycetes). Mycotaxon 134(2): 271–273. 10.5248/134.271

[B28] Magain N Sérusiaux E (2015) Dismantling the treasured flagship lichen *Stictafuliginosa* (Peltigerales) into four species in Western Europe. Mycological Progress 14(10): e97. 10.1007/s11557-015-1109-0

[B29] Mercado-Díaz JA Lücking R Moncada B Widhelm TJ Lumbsch HT (2020) Elucidating species richness in lichen fungi: The genus *Sticta* (Ascomycota: Peltigeraceae) in Puerto Rico. Taxon 69(5): 851–891. 10.1002/tax.12320

[B30] Mercado-Díaz JA Lücking R Moncada B St E Campbell KC Delnatte C Familia L Falcón-Hidalgo B Motito-Marín A Rivera-Queralta Y Widhelm TJ Lumbsch HT (2023) Species assemblages of insular Caribbean *Sticta* (lichenized Ascomycota: Peltigerales) over ecological and evolutionary time scales. Molecular Phylogenetics and Evolution 186: 107830. 10.1016/j.ympev.2023.107830 37247703

[B31] Miller MA Pfeiffer W Schwartz T (2010) Creating the CIPRES Science Gateway for Inference of Large Phylogenetic Trees. Proceedings of the Gateway Computing Environments Workshop (GCE), 14 November, 2010, New Orleans, 8 pp. 10.1109/GCE.2010.5676129

[B32] Moncada B (2012) El género *Sticta* (Schreb.) Ach. en Colombia, Taxonomía, Ecogeografía e Importancia. Doctoral thesis, Universidad Nacional de Colombia, Bogotá.

[B33] Moncada B Lücking R (2012) Ten new species of *Sticta* and counting: Colombia as a hot spot for unrecognized diversification in a conspicuous macrolichen genus. Phytotaxa 74(1): 1–29. 10.11646/phytotaxa.74.1.1

[B34] Moncada B Lücking R Betancourt-Macuase L (2013a) Phylogeny of the Lobariaceae (lichenized Ascomycota: Peltigerales), with a reappraisal of the genus *Lobariella*. Lichenologist (London, England) 45(2): 203–263. 10.1017/S0024282912000825

[B35] Moncada B Lücking R Coca LF (2013b) Six new apotheciate species of *Sticta* (lichenized Ascomycota: Lobariaceae) from the Colombian Andes. Lichenologist (London, England) 45(5): 635–656. 10.1017/S0024282913000376

[B36] Moncada B Lücking R Suárez A (2014a) Molecular phylogeny of the genus *Sticta* (lichenized Ascomycota: Lobariaceae) in Colombia. Fungal Diversity 64(1): 205–231. 10.1007/s13225-013-0230-0 24912357

[B37] Moncada B Aguirre J Lücking R (2014b) Ecogeografía del género *Sticta* (Ascomycota liquenizados: Lobariaceae) en Colombia. Revista de Biología Tropical 62(1): 266–281. 10.15517/rbt.v62i1.3564 24912357

[B38] Moncada B Suárez A Lüecking R (2015) Nueve especies nuevas del género *Sticta* (Ascomycota liquenizados: Lobariaceae) del morfotipo fuliginosa sensu lato de Colombia. Revista de la Academia Colombiana de Ciencias Exactas, Físicas y Naturales 39(150): 50–66. 10.18257/raccefyn.110

[B39] Moncada B Lücking R Lumbsch HT (2020) Rewriting the evolutionary history of the lichen genus *Sticta* (Ascomycota: Peltigeraceae subfam. Lobarioideae) in the Hawaiian islands. Plant and Fungal Systematics 65(1): 95–119. 10.35535/pfsyst-2020-0005

[B40] Moncada B Smith CW Lücking R (2021a) A taxonomic reassessment of the genus *Sticta* (lichenized Ascomycota: Peltigeraceae) in the Hawaiian archipelago. Lichenologist (London, England) 53(1): 117–133. 10.1017/S0024282920000353

[B41] Moncada B Mercado-Diáz JA Smith CW Bungartz F Sérusiaux E Lumbsch HT Lücking R (2021b) Two new common, previously unrecognized species in the *Stictaweigelii* morphodeme (Ascomycota: Peltigeraceae). Willdenowia 51(1): 35–45. 10.3372/wi.51.51103

[B42] Orange A James PW White FJ (2001) Microchemical methods for the identification of lichens. British Lichen Society, London, 101 pp.

[B43] Ossowska EA (2021) First records of *Stictaweigelii* s.str. from Bolivia confirmed by molecular data. Folia Cryptogamica Estonica 58: 65–72. 10.12697/fce.2021.58.09

[B44] Ossowska EA Moncada B Kukwa M Flakus A Rodriguez-Flakus P Olszewska S Lücking R (2022a) New species of *Sticta* (lichenised Ascomycota, lobarioid Peltigeraceae) from Bolivia suggest a high level of endemism in the Central Andes. MycoKeys 92: 131–160. 10.3897/mycokeys.92.89960 36761317 PMC9849061

[B45] Ossowska EA Kosecka M Jaskólska J Kukwa M (2022b) Two taxa of the genus *Sticta* (Peltigerales, Ascomycota), *S.andina* and S.scabrosasubsp.scabrosa, new to Bolivia confirmed by molecular data. Plant and Fungal Systematics 67(2): 45–54. 10.35535/pfsyst-2022-0006

[B46] Ossowska EA Moncada B Lücking R Flakus A Rodriguez-Flakus P Olszewska S Kukwa M (2024a) Additional new species and new records of the genus *Sticta* (lichenised Ascomycota, lobarioid Peltigeraceae) from Bolivia. MycoKeys 105: 21–47. 10.3897/mycokeys.105.120810 38694266 PMC11061559

[B47] Ossowska EA Schiefelbein U Kukwa M (2024b) First records of *Stictaarenosella* and *S.cellulosa* from South America based on molecular and morphological data. Plant and Fungal Systematics 69(1): 77–84. 10.35535/pfsyst-2024-0008

[B48] Poelt J Obermayer W (1990) Über Thallosporen bei einigen Krustenflechten. Herzogia 8(3–4): 273–288. 10.1127/herzogia/8/1990/273

[B49] Ramos D (2014) Lista de especies de líquenes y hongos liquenícolas del Perú – Checklist of lichens and lichenicolous fungi of Peru. Glalia 6(2): 1–49.

[B50] Simon A Goward T Di Meglio J Spribille T (2018a) *Stictatorii* sp. nov., a remarkable lichen of high conservation priority from northwestern North America. Graphis Scripta 30(6): 10–114.

[B51] Simon A Goffinet B Magain N Sérusiaux E (2018b) High diversity, high insular endemism and recent origin in the lichen genus *Sticta* (lichenized Ascomycota, Peltigerales) in Madagascar and the Mascarenes. Molecular Phylogenetics and Evolution 122: 15–28. 10.1016/j.ympev.2018.01.012 29360617

[B52] Simon A Lücking R Moncada B Mercado-Díaz JA Bungartz F da Silva Cáceres ME Gumboski EL de Azevedo Martins SM Spielmann AA Parker D Goffinet B (2020) *Emmanuelia*, a new genus of lobarioid lichen-forming fungi (Ascomycota: Peltigerales): Phylogeny and synopsis of accepted species. Plant and Fungal Systematics 65(1): 76–94. 10.35535/pfsyst-2020-0004

[B53] Simon A Goffinet B Wang LS Spribille T Goward T Pystina T Semenova N Stepanov NV Moncada B Lücking R Magain N Sérusiaux E (2022) Global phylogeny and taxonomic reassessment of the lichen genus *Dendriscosticta* (Ascomycota: Peltigerales). Taxon 71(2): 256–287. 10.1002/tax.12649

[B54] Stamatakis A (2014) RAxML version 8: A tool for phylogenetic analysis and post-analysis of large phylogenies. Bioinformatics (Oxford, England) 30(9): 1312–1313. 10.1093/bioinformatics/btu033 24451623 PMC3998144

[B55] Torres JM Barbosa TD Kitaura MJ Spielmann AA Lorenz AP (2021) Two new species of *Sticta* (Peltigeraceae subfam. Lobarioideae) from the Brazilian Cerrado (Brazilian savanna). The Bryologist 124(4): 506–521. 10.1639/0007-2745-124.4.506

[B56] Widhelm TJ Bertoletti FR Asztalos MJ Mercado-Díaz JA Huang JP Moncada B Lücking R Magain N Sérusiaux E Goffinet B Crouch N Mason-Gamer R Lumbsch HT (2018) Oligocene origin and drivers of diversification in the genus *Sticta* (Lobariaceae, Ascomycota). Molecular Phylogenetics and Evolution 126: 58–73. 10.1016/j.ympev.2018.04.006 29656104

[B57] Widhelm TJ Grewe F Huang JP Mercado-Díaz JA Goffinet B Lücking R Moncada B Mason-Gamer R Lumbsch HT (2019) Multiple historical processes obscure phylogenetic relationships in a taxonomically difficult group (Lobariaceae, Ascomycota). Scientific Reports 9(1): 8968. 10.1038/s41598-019-45455-x 31222061 PMC6586878

[B58] Widhelm TJ Grewe F Huang JP Ramanauskas K Mason-Gamer R Lumbsch HT (2021) Using RADseq to understand the circum-Antarctic distribution of a lichenized fungus, *Pseudocyphellariaglabra*. Journal of Biogeography 48(1): 78–90. 10.1111/jbi.13983

[B59] Yánez-Ayabaca A Benítez Á Molina RB Naranjo D Etayo J Prieto M Cevallos G Caicedo E Scharnagl K McNerlin B Swanson S Aragón G Fernández-Prado N Martínez I Burgaz AR González Y Déleg J Vega M Van Den Boom P Magain N Nugra F Oña T Díaz PJ Villalba-Alemán J Moncada B Hernández J Gilbert EE Bungartz F (2023) Towards a dynamic checklist of lichen-forming, lichenicolous and allied fungi of Ecuador - using the Consortium of Lichen Herbaria to manage fungal biodiversity in a megadiverse country. Lichenologist (London, England) 55(5): 203–222. 10.1017/S0024282923000476

